# A nonlinear relationship between the triglycerides to high-density lipoprotein cholesterol ratio and stroke risk: an analysis based on data from the China Health and Retirement Longitudinal Study

**DOI:** 10.1186/s13098-024-01339-3

**Published:** 2024-04-27

**Authors:** Shike Zhang, Changchun Cao, Yong Han, Haofei Hu, Xiaodan Zheng

**Affiliations:** 1 Department of Rehabilitation, Shenzhen Yantian District People’s Hospital, Shenzhen, 518000 Guangdong China; 2 Department of Rehabilitation, Southern University of Science and Technology Yantian Hospital, Shenzhen, 518000 Guangdong China; 3 Department of Rehabilitation, Shenzhen Second People’s Hospital, Shenzhen Dapeng New District Nan’ao People’s Hospital, Shenzhen, 518000 Guangdong China; 4 Department of Emergency, The First Affiliated Hospital of Shenzhen University, Shenzhen Second People’s Hospital, Shenzhen, 518035 Guangdong China; 5 Department of Nephrology, The First Affiliated Hospital of Shenzhen University, Shenzhen Second People’s Hospital, No. 3002, Sungang West Road, Futian District, Shenzhen, 518000 Guangdong China; 6 Department of Neurology, Shenzhen Samii Medical Center (The Fourth People’s Hospital of Shenzhen), No. 1, Jinniu West Road, Shijing Street, Pingshan District, Shenzhen, 518000 Guangdong China

**Keywords:** Nonlinearity, Triglycerides-to-high-density lipoprotein cholesterol ratio, High-density lipoprotein cholesterol, Triglycerides, Stroke

## Abstract

**Objective:**

The connection between triglycerides to high-density lipoprotein cholesterol (TG/HDL-C) ratio and stroke risk is controversial. Our goal was to explore this relationship in individuals aged 45 and older enrolled in the China Health and Retirement Longitudinal Study (CHARLS).

**Methods:**

Our analysis encompassed 10,164 participants from the CHARLS cohorts. We applied the Cox proportional-hazards regression model to evaluate the potential correlation between the TG/HDL-C ratio and stroke incidence. Using a cubic spline function and smooth curve fitting within the Cox model allowed us to unearth a possible non-linear pattern in this relationship. We also conducted thorough sensitivity and subgroup analyses to deepen our understanding of the TG/HDL-C ratio’s impact on stroke risk.

**Results:**

Adjusting for various risk factors, we observed a significant link between the TG/HDL-C ratio and increased stroke risk in individuals aged 45 and above (HR: 1.03, 95% CI 1.00–1.05, P = 0.0426). The relationship appeared non-linear, with an inflection at a TG/HDL-C ratio of 1.85. Ratios below this threshold indicated a heightened stroke risk (HR: 1.28, 95% CI 1.06–1.54, P = 0.0089), while ratios above it did not show a significant risk increase (HR: 1.01, 95% CI 0.98–1.04, P = 0.6738). Sensitivity analysis confirmed the robustness of these findings. Notably, non-smokers exhibited a stronger correlation between the TG/HDL-C ratio and stroke risk compared to past and current smokers.

**Conclusion:**

Our investigation revealed a significant, yet non-linear, association between the TG/HDL-C ratio and the incidence of stroke among individuals aged 45 and above. Specifically, we found that stroke risk increased in correlation with TG/HDL-C ratio below the threshold of 1.85. These insights may guide healthcare providers in advising and developing more effective strategies for stroke prevention in this demographic.

**Supplementary Information:**

The online version contains supplementary material available at 10.1186/s13098-024-01339-3.

## Background

As a common cause of disability and death globally, stroke carried a significant financial burden for treatment and post-stroke healthcare [1]. According to the epidemiological statistics on the worldwide burden of illness, stroke caused 6.55 million deaths and 143 million disability-adjusted life years in 2019 [2]. The elevated prevalence of stroke remained a substantial financial and healthcare burden. Previous research has elucidated a significant association between the prevalence of chronic conditions such as diabetes mellitus, hypertension, cardiovascular diseases, dyslipidemia, and chronic kidney disease and an increased risk of stroke among individuals in the middle-aged and elderly cohorts [3, 4]. Nevertheless, it is important to note that conventional risk factors alone are insufficient in comprehensively elucidating all the potential risks associated with stroke [5, 6].

Dyslipidemia, characterized by abnormal levels of lipids in the blood, is often evidenced by diminished high-density lipoprotein cholesterol (HDL-C) and increased low-density lipoprotein cholesterol (LDL-C) and triglycerides (TG). Recognized as a key risk factor in stroke development [7], recent studies have shifted focus to less traditional lipid indices, such as the ratio of triglycerides to high-density lipoprotein cholesterol (TG/HDL-C) ratio. This ratio has gained recognition as a significant marker in assessing the risk for cardiovascular diseases and metabolic syndrome [8, 9]. Although a high TG/HDL-C ratio has been linked to a greater risk of stroke [10, 11], there have been studies with conflicting results [12, 13]. This appearance may be due to differences in the TG/HDL-C ratio range, the sample sizes, and adjustment variables. Moreover, nonlinear relationships may alter linear regression study conclusions, resulting in changes in the fitted linear relationship. Therefore, we analyzed the data using the China Health and Retirement Longitudinal Study (CHARLS) to observe the relationship between the TG/HDL-C ratio and stroke risk.

## Methods

### Data source

In this longitudinal research, the dataset was derived from the CHARLS, covering a period from 2011 to 2018. The study’s sample was drawn from a broad cross-section of the population, encompassing 450 communities within 150 counties across 28 provinces. Utilizing a systematic methodology, CHARLS gathered biennial data encompassing demographic characteristics, nutritional habits, and overall health statuses of residents, family units, and larger community settings. In the baseline survey in 2011–2012, we included 17,708 participants (Wave 1). Data on exposure variables and all covariates were measured in 2011–2012. Participants received three follow-up visits in 2013–2014 (Wave 2), 2015–2016 (Wave 3), and 2017–2018 (Wave 4). Our study included participants if they attended any of the Wave 2, Wave 3, and Wave 4 follow-ups.

### Study population

Authorization for conducting the CHARLS was officially obtained from the Biomedical Ethics Review Board of Peking University, with all subjects providing written consent in accordance with ethical standards [14]. Our study was conducted in strict alignment with the ethical guidelines set forth by the Declaration of Helsinki, ensuring compliance with all relevant regulations and legal requirements during the research process.

**Fig. 1:**
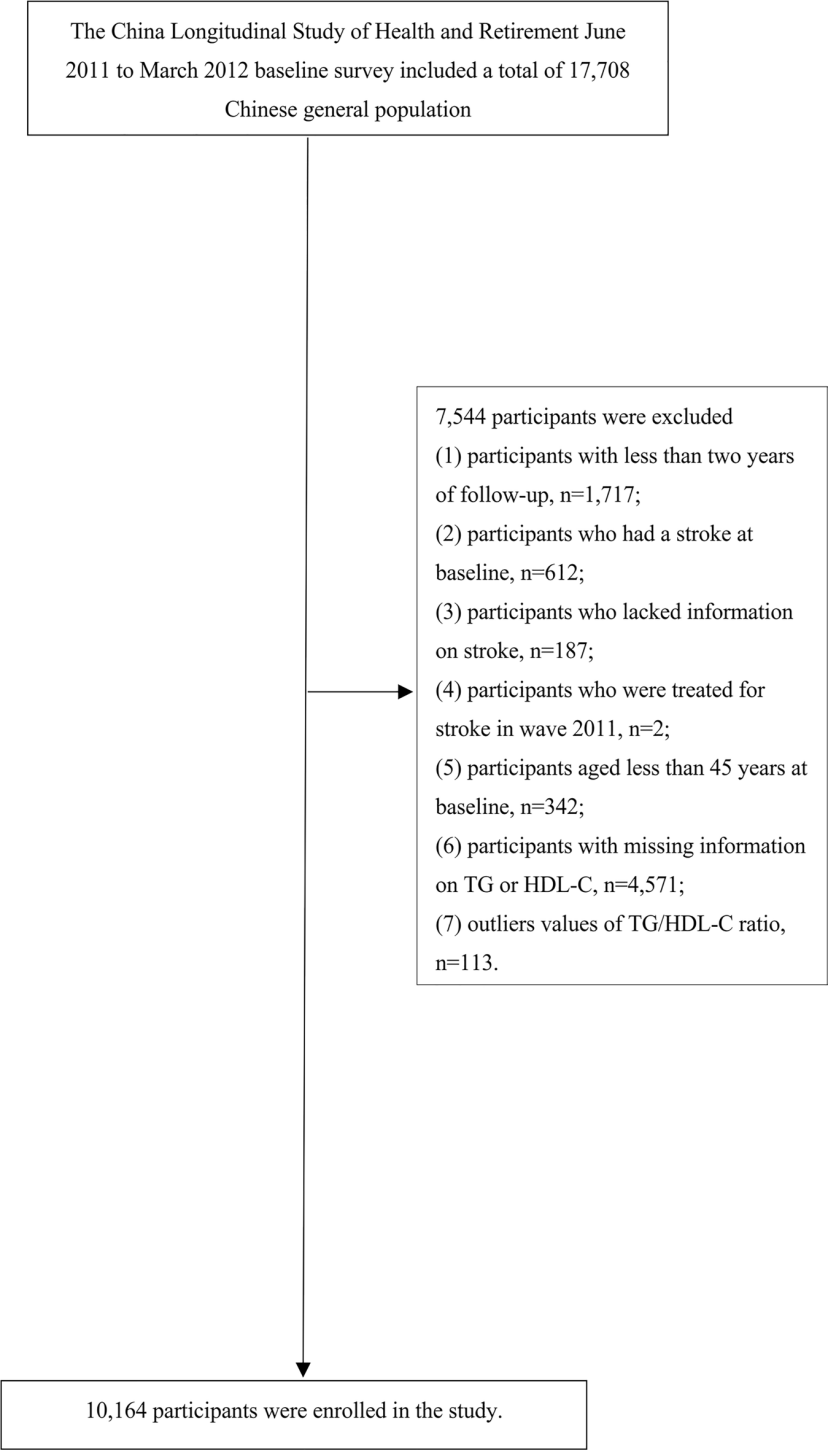
Study population

Criteria for exclusion from the participant pool included individuals with less than 2 years of follow-up, a pre-existing stroke condition, incomplete stroke data, being treated or having been treated for stroke at baseline, those under the age of 45, missing baseline data for HDL-C or TG, and TG/HDL-C ratios that were outliers (beyond three standard deviations from the mean). After applying these criteria, the cohort for our final analysis was comprised of 10,164 subjects. The methodology and flow of participants through the study are depicted in Fig. 1.

### TG/HDL-C ratio

Serum TG level (mg/dL) divided by HDL-C level (mg/dL) was used to compute the TG/HDL-C ratio.

### Diagnosis of stroke

In our study, the outcome of interest in this study was the occurrence of stroke during the follow-up period. We identified incident cases as participants who, being devoid of stroke at the commencement of the study, subsequently reported experiencing a stroke during the follow-up assessments. Data pertaining to the occurrence of stroke were meticulously collected through a structured self-reported questionnaire [14, 15], designed to elicit comprehensive information on three critical aspects: (1) Were you informed of a stroke diagnosis by a medical professional? (2) When did you initially receive or become aware of the diagnosis? (3) Do you have any therapy for your stroke at this time? Affirmative responses during follow-up were classified as first-time stroke diagnoses, with the reported date marking the onset. The interval between the stroke onset and baseline assessment was calculated to establish the timing of the stroke. For those without reported strokes during follow-up, we determined follow-up duration by the interval between the baseline assessment and their final survey date.

### Data collection

Healthcare professionals and skilled surveyors collected a range of data from participants, including demographic details, body measurements, and health indicators. Systolic blood pressure (SBP), age, height, drinking habits, sex, diastolic blood pressure (DBP), smoking habits, weight and physical activity were all recorded. Physical activity was defined as engaging in either moderate-intensity exercise for at least 2.5 h weekly, vigorous exercise for a minimum of 1.25 h weekly, or a combination that equates to 600 or more metabolic equivalent minutes each week [16]. Smoking status was categorized into three distinct groups according to individuals’ smoking behavior: current smokers, individuals who have smoked in the past, and individuals who have never smoked. Similarly, drinking status was classified into three categories based on individuals’ drinking behavior: current drinkers, individuals who have previously consumed alcohol, and individuals who have never consumed alcohol. Weight/Height^2^ (kg/m^2^) was used to compute BMI. Hypertension was defined as SBP ≥ 140 mmHg, DBP ≥ 90 mmHg (average of 3 measurements), or hypertension history. Blood samples were taken by the medical staff from the Chinese Center for Disease Control and Prevention after an overnight fast. These samples were analyzed in a central laboratory to measure various biomarkers, including total cholesterol (TC), glycosylated hemoglobin (HbA1c), C-reactive protein (CRP), LDL-C, serum creatinine (Scr), HDL-C, serum cystatin C, TG, fasting plasma glucose (FPG), and uric acid (UA).

### Missing data processing

The dataset used in the study exhibited missing values for several clinical variables, constituting a small fraction of the overall dataset. Specifically, missing values were observed for gender in 8 individuals (0.08%), BMI in 1503 individuals (14.62%), DBP in 1463 individuals (14.24%), hypertension in 55 individuals (0.54%), drinking status in 11 individuals (0.11%), SBP in 1463 individuals (14.24%), physical activity in 6011 individuals (58.49%), smoking status in 169 individuals (1.64%), FPG in 14 individuals (0.14%), TC in 3 individuals (0.03%), CRP in 1 individual (0.01%), LDL-C in 21 individuals (0.20%), Scr in 18 individuals (0.18%), HbA1c in 80 individuals (0.78%), Cystatin C in 2472 individuals (24.05%), and UA in 1 individual (0.01%). To address the missing clinical variables, multiple imputations via chained equations were employed for modeling purposes. The imputation model incorporated the following variables: smoking status, DBP, SBP, gender, physical activity, drinking status, age, hypertension, drugs for cardiovascular prevention, BMI, FPG, TC, Scr, CRP, LDL-C, Cystatin C HbA1c, and UA. The analysis of missing data followed the assumption of missing-at-random to ensure the validity of the imputation process [17].

### Statistical analysis

Statistical computations were performed utilizing the R software environment along with Empower Stats. The initial categorization of baseline characteristics segmented the dataset into four groups based on quartiles of the TG/HDL-C ratio. We presented categorical variables using frequency counts and percentages, whereas median and interquartile ranges (25th–75th percentile) or mean values with standard deviations (SD) were used for continuous variables. To examine differences among the four groups, we utilized the Kruskal–Wallis H test for data with a skewed distribution, the One-Way ANOVA test for normally distributed data, or the χ^2^ test for categorical data.

We constructed multivariate Cox proportional hazards regression models in a three-tiered approach to test the correlation between the TG/HDL-C ratio and stroke: (1) Model I: this Model did not incorporate any covariates; (2) Model II: this Model adjusted for socio-demographic factors, including smoking status, gender, physical activity, drinking status, age, and BMI; (3) Model III: this Model adjusted for all factors, including smoking status, gender, physical activity, drinking status, age, BMI, hypertension, drugs for cardiovascular prevention, FPG, TC, Scr, CRP, LDL-C, Cystatin C HbA1c, and UA. We reported both adjusted and unadjusted hazard ratios (HR) with their 95% confidence intervals (CI).

The sensitivity analyses were conducted to check the validity of our findings. We categorized the TG/HDL-C ratio into groups based on its quartile distribution. We then determined the P-value for the trend to evaluate the significance of the TG/HDL-C ratio when considered a continuous variable and to examine its potential non-linear relationship with stroke risk. Because hypertension and drugs for cardiovascular prevention may influence the relationship between TG/HDL-C and stroke, we performed additional sensitivity analyses by excluding individuals with hypertension or drugs for cardiovascular prevention to investigate the connection between the TG/HDL-C ratio and stroke in subgroups.

To evaluate the non-linear relationship between the TG/HDL-C ratio and the occurrence of stroke, we utilized the Cox proportional hazards regression model, incorporating cubic spline functions and smooth curve fitting techniques. Upon detection of a non-linear correlation, we pinpointed the inflection point through recursive techniques. Subsequently, we applied a two-piecewise Cox proportional hazards regression model to each segment divided by the inflection point. The determination of the most suitable model to clarify the link between the TG/HDL-C ratio and stroke risk relied on the outcomes of a log-likelihood ratio test.

The subgroup analysis was performed through the utilization of the Cox proportional hazard model. The following variables were converted into categorical variables: BMI (< 25 kg/m^2^, ≥ 25 kg/m^2^) and age (< 65 years, ≥ 65 years) according to clinical cutoffs. With the exception of the stratification variable, each stratification was given a fully adjusted analysis. The likelihood ratio test was performed to validate the interactions between subgroups. All results follow the STROBE statement [18]. Statistical significance was established using a two-tailed test with a threshold of P < 0.05.

## Results

### Characteristics of individuals

This investigation included a cohort of 10,164 individuals without any history of stroke at baseline. The average age was 59.18 ± 9.35 years. 1,191 (11.72%) individuals developed stroke during follow-up.

The population’s baseline characteristics are shown in Table 1. Based on quartiles of the TG/HDL-C ratio (Q1 ≤ 1.32; 1.32 < Q2 ≤ 2.11; 2.11 < Q3 ≤ 3.56; Q4 > 3.56), all subjects were classified into four groups. Participants had higher levels of DBP, FPG, TG, BMI, SBP, TC, CRP, HbA1c, Scr, and UA in the Q4 group. There were higher rates of hypertension, drugs for cardiovascular prevention, ever drinkers and ever smokers in the Q4 group. A lower level of HDL-C and LDL-C was observed in the Q1 group. Participants had higher rates of physical activity and males in the Q1 group.

**Table 1:** The baseline characteristics of participants

TG/HDL-C ratio	Q1 (≤ 1.32)	Q2 (1.32 to ≤ 2.11)	Q3 (2.11 to ≤ 3.56)	Q4 (> 3.56)	P-value
Participants	2539	2542	2542	2541	
Gender					< 0.001^b^
Male	1317 (51.87%)	1173 (46.14%)^a^	1126 (44.30%)^a^	1130 (44.47%)^a^	
Female	1222 (48.13%)	1369 (53.86%)	1416 (55.70%)	1411 (55.53%)	
Age (years)	59.89 ± 9.76	59.32 ± 9.48^a^	59.04 ± 9.11^a^	58.45 ± 8.96^a^	< 0.001^d^
Drinking status					< 0.001^b^
Never drinkers	312 (12.29%)	350 (13.77%)^a^	378 (14.87%)^a^	344 (13.54%)^a^	
Ever drinkers	1398 (55.06%)	1580 (62.16%)	1609 (63.30%)	1633 (64.27%)	
Current drinkers	829 (32.65%)	612 (24.08%)	555 (21.83%)	564 (22.20%)	
Smoking status					< 0.001^b^
Never smokers	1469 (57.86%)	1571 (61.80%)^a^	1605 (63.14%)^a^	1605 (63.16%)^a^	
Ever smokers	221 (8.70%)	204 (8.03%)	217 (8.54%)	240 (9.45%)	
Current smokers	849 (33.44%)	767 (30.17%)	720 (28.32%)	696 (27.39%)	
Physical activity					< 0.001^b^
No	790 (31.11%)	896 (35.25%)^a^	945 (37.18%)^a^	962 (37.86%)^a^	
Yes	1749 (68.89%)	1646 (64.75%)	1597 (62.82%)	1579 (62.14%)	
Drugs for cardiovascular prevention					< 0.001^b^
No	2379 (93.70%)	2359 (92.80%)	2338 (91.97%)^a^	2280 (89.73%)^a^	
Yes	160 (6.30%)	183 (7.20%)	204 (8.03%)	261 (10.27%)	
Hypertension					< 0.001^b^
No	1678 (66.09%)	1589 (62.51%)^a^	1409 (55.43%)^a^	1249 (49.15%)^a^	
Yes	861 (33.91%)	953 (37.49%)	1133 (44.57%)	1292 (50.85%)	
SBP (mmHg)	128.03 ± 21.01	129.23 ± 20.94^a^	131.77 ± 21.70^a^	133.32 ± 21.31^a^	0.011^d^
DBP (mmHg)	73.98 ± 12.04	74.83 ± 11.71^a^	76.45 ± 12.15^a^	77.98 ± 12.01^a^	< 0.001^d^
BMI (kg/m^2^)	22.08 ± 3.42	22.98 ± 3.83^a^	24.00 ± 3.95^a^	25.04 ± 3.89^a^	< 0.001^d^
TG/HDL-C ratio	0.96 ± 0.23	1.69 ± 0.23^a^	2.73 ± 0.41^a^	6.41 ± 3.13^a^	< 0.001^d^
HDL-C (mg/dL)	67.26 ± 14.34	54.13 ± 10.12^a^	46.88 ± 8.66^a^	37.56 ± 7.91^a^	< 0.001^d^
TG (mg/dL)	62.59 ± 15.09	91.13 ± 18.44^a^	127.07 ± 26.02^a^	229.07 ± 89.30^a^	< 0.001^d^
LDL-C (mg/dL)	111.06 ± 29.89	119.04 ± 32.66^a^	123.42 ± 34.41^a^	113.94 ± 37.98^a^	< 0.001^d^
TC (mg/dL)	188.07 ± 34.74	189.16 ± 36.70	194.14 ± 37.53^a^	200.17 ± 41.83^a^	< 0.001^d^
CRP (mg/L)	0.75 (0.44–1.74)	0.91 (0.52–1.88)^a^	1.08 (0.58–2.25)^a^	1.32 (0.72–2.63)^a^	< 0.001^c^
Scr (mg/dL)	0.78 ± 0.32	0.77 ± 0.22	0.78 ± 0.20	0.80 ± 0.20^a^	< 0.001^d^
FPG (mg/dL)	102.23 ± 23.48	105.34 ± 28.41^a^	109.09 ± 34.61^a^	120.99 ± 45.84^a^	< 0.001^d^
HbA1c (%)	5.13 ± 0.61	5.19 ± 0.70^a^	5.25 ± 0.82^a^	5.40 ± 0.98^a^	< 0.001^d^
Cystatin C (mg/L)	1.01 ± 0.31	1.03 ± 0.27	1.01 ± 0.27	0.96 ± 0.26^a^	< 0.001^d^
UA (mg/dL)	4.24 ± 1.16	4.32 ± 1.22^a^	4.45 ± 1.24^a^	4.79 ± 1.31^a^	< 0.001^d^

Values are n (%) or mean ± SD or median (quartile)

*TG/HDL-C ratio* ratio of triglyceride to high-density lipoprotein cholesterol, *SBP* systolic blood pressure, *DBP* diastolic blood pressure, *BMI* body mass index, *HDL-C* high-density lipoprotein cholesterol, *LDL-C* low-density lipoprotein cholesterol, *TC* total cholesterol, *TG* triglycerides, *Scr* serum creatinine, *CRP* C-reactive protein, *FPG* fasting plasma glucose, *HbA1c* glycosylated hemoglobin, *UA* uric acid

^a^Represents other groups (Q2, Q3, Q4) compared with Q1, P < 0.05

^b^Used the χ^2^ test

^c^Applied the Kruskal–Wallis

^d^Used the one-way ANOVA test

### The incidence rate of stroke

Table 2 presents the stroke incidence rates observed in a cohort of 10,164 individuals during the follow-up period. The overall population’s cumulative incidence rate of stroke was 11.72% (11.09–12.34%). The cumulative incidence rates of four TG/HDL-C ratio groups were 8.63% (7.53–9.72%), 10.78% (9.57–11.99%), 12.63% (11.34–13.92%) and 14.84% (13.45–16.22%), respectively. The total incidence rate of all people, Q1 groups, Q2 groups, Q3 groups, and Q4 groups were 19.21, 13.94, 17.64, 20.72, and 24.69 per 1000 person-years, respectively. Subjects in the Q4 group exhibited a significantly higher stroke incidence rate compared to those in the Q1 group (P < 0.001 for trend).

**Table 2:** Incidence rate of incident stroke

TG/HDL-C ratio	Participants (n)	Stroke events (n)	Incidence rate (95% CI) (%)	Per 1000 person-year
Total	10,164	1191	11.72 (11.09–12.34)	19.21
Q1	2539	219	8.63 (7.53–9.72)	13.94
Q2	2542	274	10.78 (9.57–11.99)	17.64
Q3	2542	321	12.63 (11.34–13.92)	20.72
Q4	2541	377	14.84 (13.45–16.22)	24.69
P for trend			< 0.001	< 0.001

*TG/HDL-C ratio* triglyceride to high-density lipoprotein cholesterol ratio, *CI* confidence interval

### The results of the correlation between the TG/HDL-C ratio and stroke

As the TG/HDL-C ratio met the proportional hazards assumption, the association between the TG/HDL-C ratio and stroke risk was evaluated by the Cox proportional hazards regression model. Table 3 shows the correlation between TG/HDL-C ratio and stroke based on Cox proportional hazards regression models. In Model I, the TG/HDL-C ratio exhibited a positive association with stroke (HR: 1.06, 95% CI 1.04–1.08, P < 0.0001). The HR in the Model II and Model III were 1.04 (1.02–1.06) and 1.03 (1.00–1.05), respectively. After accounting for all relevant factors, the results demonstrated that each additional unit of the TG/HDL-C ratio was found to be associated with only a 3% increase in stroke risk.

**Table 3:** Relationship between TG/HDL-C ratio and the incident stroke in different models

Exposure	Model I (HR, 95% CI, P)	Model II (HR, 95% CI, P)	Model III (HR, 95% CI, P)
TG/HDL-C ratio	1.06 (1.04, 1.08) < 0.0001	1.04 (1.02, 1.06) < 0.0001	1.03 (1.00, 1.05) 0.0426
TG/HDL-C ratio (quartile)			
Q1	Ref	Ref	Ref
Q2	1.27 (1.06, 1.51) 0.0089	1.21 (1.01, 1.44) 0.0385	1.17 (0.98, 1.40) 0.0912
Q3	1.48 (1.25, 1.76) < 0.0001	1.36 (1.14, 1.61) 0.0006	1.25 (1.04, 1.49) 0.0154
Q4	1.77 (1.49, 2.09) < 0.0001	1.57 (1.32, 1.86) < 0.0001	1.32 (1.10, 1.58) 0.0023
P for trend	< 0.0001	< 0.0001	0.0021

Model I: we did not adjust for other covariants

Model II: we adjusted for BMI, gender, age, drinking status, physical activity, and smoking status

Model III: we adjusted for BMI, gender, age, drinking status, physical activity, smoking status, drugs for cardiovascular prevention, hypertension, FPG, TC, CRP, LDL-C, Scr, HbA1c, Cystatin C, and UA

*HR* hazard ratios, *CI* confidence interval, *Ref* reference, *GAM* generalized additive mode, *TG/HDL-C ratio* ratio of triglyceride to high-density lipoprotein cholesterol

### Sensitivity analyses

To solidify the credibility of our research outcomes, multiple sensitivity checks were performed. The TG/HDL-C metric was recategorized for inclusion in our model. The analysis revealed inconsistent effect size trends across categories, hinting at a possible non-linear link with stroke events. In our research, we also ran sensitivity checks on participants without hypertension. After adjusting for various confounders, the data still showed a significant link between the TG/HDL-C ratio and the risk of stroke (HR = 1.05, 95% CI 1.01–1.09, P = 0.0170) as indicated in Table 4. Moreover, we carried out additional sensitivity analyses on individuals without drugs for cardiovascular prevention. These analyses also supported the positive relationship between the TG/HDL-C ratio and stroke risk, even after adjustments for confounders (HR = 1.03, 95% CI 1.00–1.06, P = 0.0263), as shown in Table 4.

**Table 4:** Relationship between TG/HDL-C ratio and stroke in different sensitivity analyses

Exposure	Model I (HR, 95% CI, P)	Model II (HR, 95% CI, P)
TG/HDL-C ratio	1.05 (1.01, 1.09) 0.0170	1.03 (1.00, 1.06) 0.0263
TG/HDL-C ratio (Quintile)		
Q1	Ref	Ref
Q2	1.12 (0.86, 1.46) 0.4108	1.14 (0.93, 1.41) 0.2152
Q3	1.31 (1.00, 1.71) 0.0470	1.26 (1.03, 1.55) 0.0261
Q4	1.48 (1.13, 1.95) 0.0047	1.34 (1.09, 1.65) 0.0062
P for trend	0.0023	0.0039

Model I was sensitivity analysis in participants without hypertension. We adjusted BMI, gender, age, drinking status, physical activity, smoking status, drugs for cardiovascular prevention, FPG, TC, CRP, LDL-C, Scr, HbA1c, Cystatin C, and UA

Model II was sensitivity analysis in participants without drugs for cardiovascular prevention. We adjusted BMI, gender, age, drinking status, physical activity, smoking status, hypertension, FPG, TC, CRP, LDL-C, Scr, HbA1c, Cystatin C, and UA

*HR* hazard ratios, *CI* confidence, *Ref* reference, *TG/HDL-C ratio* ratio of triglyceride to high-density lipoprotein cholesterol

### The analysis of the nonlinear relationship

**Fig. 2:**
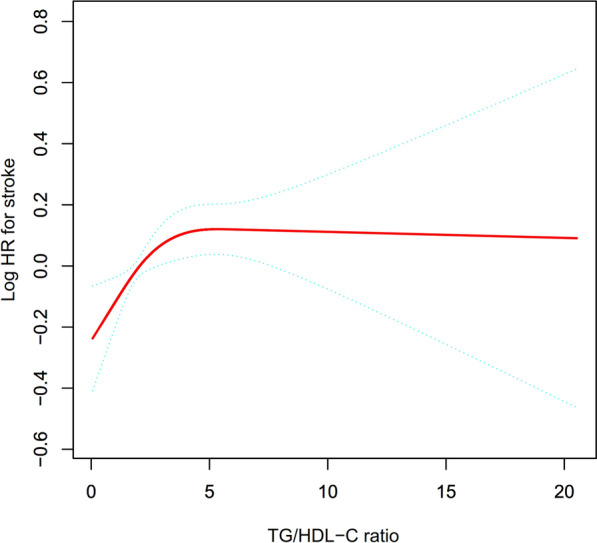
The nonlinear relationship between TG/HDL-C ratio and incident stroke in Chinese individuals aged 45 years or older. A nonlinear relationship between the TG/HDL-C ratio and stroke was detected after adjustment for BMI, gender, age, drinking status, physical activity, smoking status, drugs for cardiovascular prevention, hypertension, FPG, TC, CRP, LDL-C, Scr, HbA1c, Cystatin C, and UA

Figure 2 illustrates the non-linear correlation between the TG/HDL-C ratio and stroke incidence. A non-linear association was confirmed after adjusting for a comprehensive set of variables (Table 5). The analysis identified an inflection point in the TG/HDL-C ratio at 1.85 using a two-piecewise Cox proportional hazards regression model. Below this inflection point, an increased likelihood of stroke was associated with the TG/HDL-C ratio (HR: 1.28, 95% CI 1.06–1.54, P = 0.0089). Above this ratio, however, the association did not reach statistical significance (HR: 1.01, 95% CI 0.98–1.04, P = 0.6738).

**Table 5:** The result of the two-piecewise Cox proportional hazards regression model

Incident stroke	HR (95% CI)	P
Fitting model by standard Cox regression	1.03 (1.00, 1.05)	0.0426
Fitting model by two-piecewise Cox proportional hazards regression model		
The inflection point of TG/HDL-C ratio		
≤ 1.85	1.28 (1.06, 1.54)	0.0089
> 1.85	1.01 (0.98, 1.04)	0.6738
P for the log-likelihood ratio test	0.016	

We adjusted for BMI, gender, age, drinking status, physical activity, smoking status, drugs for cardiovascular prevention, hypertension, FPG, TC, CRP, LDL-C, Scr, HbA1c, Cystatin C, and UA

*HR* hazard ratios, *CI* confidence, *TG/HDL-C ratio* ratio of triglyceride to high-density lipoprotein cholesterol

### Subgroup analysis

As shown in Table 6, the potential confounding variables that might have affected the correlation between the TG/HDL-C ratio and stroke were found in subgroup analysis. Stratification was conducted based on pertinent variables: smoking status, gender, physical activity, drinking status, age, and BMI. With the exception of smoking status, none of the confounding variables had an effect on the correlation between the TG/HDL-C ratio and stroke (P = 0.0331). The relationship was more significant in individuals with never smokers.

**Table 6:** Effect size of TG/HDL-C ratio on stroke in prespecified and exploratory subgroups

Characteristic	No of participants	HR (95% CI) P value	P for interaction
Age (years)			0.7958
< 65	7453	1.02 (0.99, 1.05) 0.1153	
≥ 65	2711	1.03 (0.99, 1.07) 0.1305	
Gender			0.3896
Male	4746	1.04 (1.00, 1.07) 0.0294	
Female	5418	1.02 (0.99, 1.05) 0.2225	
BMI (kg/m^2^)			0.8826
< 25	6936	1.03 (1.00, 1.06) 0.0791	
≥ 25	3228	1.03 (0.99, 1.06) 0.1257	
Smoking status			0.0331
Never smokers	6250	1.05 (1.01, 1.08) 0.0047	
Ever smokers	882	1.02 (0.96, 1.09) 0.4545	
Current smokers	3032	0.98 (0.94, 1.03) 0.4822	
Drinking status			0.5347
Never drinkers	1384	0.99 (0.93, 1.06) 0.8557	
Ever drinkers	6220	1.05 (1.02, 1.09) 0.0010	
Current drinkers	2560	0.99 (0.94, 1.04) 0.5865	
Physical activity			0.4156
No	3593	1.03 (1.00, 1.07) 0.0424	
Yes	6571	1.02 (0.99, 1.05) 0.2286	

The above model was adjusted for BMI, gender, age, drinking status, physical activity, smoking status, drugs for cardiovascular prevention, hypertension, FPG, TC, CRP, LDL-C, Scr, HbA1c, Cystatin C, and UA

The model is not adjusted for the stratification variable in each case

## Discussion

Our study supported that the TG/HDL-C ratio was positively associated with stroke incidence. An inflection point was also identified, and different connections between the TG/HDL-C ratio and stroke risk were observed on either side of this point. The correlation between the TG/HDL-C ratio and stroke was more significant in individuals with never smokers.

Non-traditional lipid profiles, such as non-high-density lipoprotein cholesterol, LDL-C to HDL-C ratio, TC to HDL-C ratio, and TG/HDL-C ratio, have recently drawn increasing attention. The development of the metabolic syndrome is closely correlated with non-traditional lipid parameters, particularly the TG/HDL-C ratio. TG/HDL-C ratio was regarded as a prognostic marker of insulin resistance that may increase the risk of metabolic syndrome more quickly than single lipid measurements [19]. However, the question of whether the TG/HDL-C ratio raises stroke risk is still up for debate. In a prospective study involving 96,542 Chinese individuals, the high TG/HDL-C ratio group was an independent risk factor for stroke compared with the low TG/HDL-C ratio group after controlling for confounding variables (HR:1.11, 95% CI 1.03–1.18) [10]. Another study from China involving 5099 hypertensive patients showed that the TG/HDL-C ratio was strongly associated with future stroke after adjusting for ethnicity, current smoking, sex, age, BMI, diabetes, DBP, heavy drinking, SBP, and anti-hypertension drug treatment (HR: 1.58, 95% CI 1.21–2.06) [7]. Existing literature has also substantiated that the TG/HDL-C ratio serves as a more discerning biomarker for the identification of insulin resistance and the prognostication of diabetes, gestational diabetes, and cardiovascular incidents when compared to the unconventional lipid parameters, such as LDL-C to HDL-C ratio, and TC to HDL-C ratio [20–24]. In addition, previous studies have demonstrated that Lifestyle interventions and pharmacotherapy that target the reduction of triglycerides and the elevation of HDL-C have been shown to improve the TG/HDL-C ratio, thereby enhancing insulin sensitivity and reducing the risk of atherosclerosis, cardiovascular disease, and stroke risk [25–27]. However, a prospective cohort study from Japan, including 11,699 individuals, demonstrated that the TG/HDL-C ratio was not associated with stroke risk in the overall population (HR: 1.28, 95% CI 0.94–1.75) after controlling for age, hypertension, BMI, smoking status, diabetes mellitus, TC, and alcohol consumption [13]. In another prospective cohort study that included 2940 residents who did not have a stroke in the northern Manhattan, New York, the results showed that the TG/HDL-C ratio was not linked to the risk of stroke after adjusting for confounders (HR: 0.98, 95% CI 0.92–1.04) [12]. Our results supported the idea that the high TG/HDL-C ratio increases new-onset stroke risk. This may be due to differences in the TG/HDL-C ratio range, the sample sizes, and different adjustment variables. Moreover, nonlinear relationships may alter linear regression study conclusions, resulting in changes in the fitted linear relationship. Hence, the TG/HDL-C ratio may be non-linearly associated with incident stroke, explaining the disparity in these studies’ results. On the other hand, the sensitivity analysis discovered that a positive association still exists among those without hypertension or drugs for cardiovascular prevention. These findings serve as a reference point for improving therapies that aim to reduce the individuals aged 45 years or older risk of developing stroke.

This study’s subgroup analysis produced some intriguing results. In comparison to other smoking status groups, individuals with never-smokers had a more significant connection between the TG/HDL-C ratio and stroke risk. Further analysis of the study population’s baseline data, which was categorized by smoking status, revealed that never smokers had lower age, DBP, CRP, Scr, Cystatin C, UA, and a smaller percentage of current drinkers (Additional file 1: Table S1). In never smokers, the levels of these risks, as mentioned above, are lower, so the impact of the TG/HDL-C ratio on stroke is strengthened.

Uncertainty surrounds the mechanism by which the TG/HDL-C ratio is linked to stroke. Nonetheless, we surmise that arteriosclerosis plays a role in pathogenesis. The TG/HDL-C ratio is closely related to metabolic syndrome [28–30], which causes a high inflammatory response and increases oxidative stress [31], leading to endothelial dysfunction and consequent atherosclerosis [32, 33]. Moreover, the small dense low-density lipoprotein cholesterol (sdLDL-C), which is linked to the pathophysiology of atherosclerosis, may be connected with the TG/HDL-C ratio. The sdLDL-C’s prolonged circulation period and small particle size facilitate their penetration into the artery wall, allowing them to store lipids and cholesterol [34]. Moreover, their propensity for oxidation may contribute to an increase in their atherogenicity [35]. The sdLDL-C levels are helpful in assessing residual coronary heart disease risk [36] and are an important indicator of incident stroke [37]. TG is transferred into LDL-C particles when the liver secretes TG-rich lipoprotein, where hepatic lipases further delipidate it to produce smaller LDL particles [33]. Moreover, hepatic lipase activity is made more active in this process when HDL-C is lower [38]. These pathways may provide an explanation for why higher TG/HDL-C levels raise the risk of stroke.

Furthermore, the current investigation discovered a nonlinear connection between the TG/HDL-C ratio and stroke risk. To comprehend non-linear correlation more effectively, this study employed a two-piecewise Cox proportional hazards regression model. The correlation between the TG/HDL-C ratio and stroke among subjects aged 45 or older demonstrated a non-linear pattern, indicating a saturation effect. Following adjustment for confounders, the TG/HDL-C ratio inflection point was identified as 1.85. The study revealed that for every unit increase in the TG/HDL-C ratio below 1.85, the risk of stroke escalated by 28% (HR: 1.28, 95% CI 1.06–1.54, P = 0.0089). However, elevating the TG/HDL-C ratio above 1.85 did not exhibit an associated increase in the likelihood of stroke (HR: 1.01, 95% CI 0.98–1.04, P = 0.6738). Our findings establish a theoretical basis supporting the management of the TG/HDL-C ratio within clinical settings, particularly emphasizing the significance when the TG/HDL-C ratio decreases to less than 1.85. Furthermore, our results provide additional insights aimed at aiding subjects across varying TG/HDL-C ratios in mitigating the risk of stroke.

We listed the following strengths of our research. Primarily, we employed multiple imputations to address missing data, thus enhancing the statistical power and alleviating potential biases stemming from the absence of covariate information. Additionally, to minimize the impact of residual confounding factors on the results, we rigorously adjusted statistically. Furthermore, we conducted sensitivity analyses to ensure the robustness of our findings. These analyses involved categorizing the TG/HDL-C ratio and reexamining the association after excluding individuals with hypertension or drugs for cardiovascular prevention. Lastly, we conducted subgroup analyses to assess other potential confounding data that might influence the correlation between the TG/HDL-C ratio and stroke.

There are certain limitations to the current investigation. First, our research recruited Chinese over 45 years of age. Therefore, these results need further validation for other ethnicities and younger groups. Second, a self-reported questionnaire used in the CHARLS trial to confirm event strokes may have introduced recollection bias and misclassification mistakes. Third, the stroke diagnosis was based on self-reported questionnaires to ascertain incident strokes, and this study did not distinguish between ischaemic and hemorrhagic strokes in the diagnosis of stroke. There are some notable differences in risk factors between ischaemic and hemorrhagic stroke [39]. In the future, we will conduct our study, and we will combine stroke-related symptoms and imaging findings to differentiate between ischaemic and hemorrhagic stroke. Fourth, some variables, such as physical activity and BMI, have substantial missing data that may affect the reliability and accuracy of the estimates. In the future, we will also design our study to reduce the missing data and make our findings reliable. Fifth, possible unmeasured confounders may have affected the association between the TG/HDL-C ratio and stroke risks, such as the family history of stroke and nutrition.

## Conclusion

Our study demonstrated a positive and nonlinear association between the TG/HDL-C ratio and stroke risk among people aged 45 or older. A statistically significant positive connection exists between the TG/HDL-C ratio and incident stroke when the TG/HDL-C ratio is lower than 1.85. Lowering the TG/HDL-C ratio, especially when it drops to less than 1.85, may reduce stroke risk in clinical practice.

### Supplementary Information

**Additional file 1: Table S1.** The characteristics of participants in smoking status. **Table S2.** Relationship between TG/HDL-C ratio and the incident stroke in different sensitivity models. **Table S3.** Relationship between TG/HDL-C ratio and the incident stroke in different sensitivity models.

## Data Availability

Data are available from http://www.isss.pku.edu.cn/cfps/. Follow the prompts to register as a user and download the data once it has been reviewed and approved.

## Abbreviations

TG/HDL-C ratio: Ratio of triglyceride to high-density lipoprotein cholesterol
CHARLS: China Health and Retirement Longitudinal Study
BMI: Body mass index
SBP: Systolic blood pressure
DBP: Diastolic blood pressure
FPG: Fasting plasma glucose
TC: Total cholesterol
TG: Triglyceride
HDL-C: High-density lipoprotein cholesterol
LDL-C: Low-density lipid cholesterol
Scr: Serum creatinine
HbA1c: Glycosylated hemoglobin
CRP: C-reactive protein
UA: Uric acid
HR: Hazard ratios
CI: Confidence intervals
Ref: Reference
sdLDL-C: Small dense low-density lipoprotein-cholesterol
