# Supplementary material for: A nonlinear relationship between the triglycerides to high-density lipoprotein cholesterol ratio and stroke risk: an analysis based on data from the China Health and Retirement Longitudinal Study
Source: Diabetol Metab Syndr. 2024 Apr 27;16:96. doi: 10.1186/s13098-024-01339-3 (PMC11055270; doi:10.1186/s13098-024-01339-3)
Supplement: Supplementary file 1 — Additional file 1: Table S1. The characteristics of participants in smoking status. Table S2. Relationship between TG/HDL-C ratio and the incident stroke in different sensitivity models. Table S3. Relationship between TG/HDL-C ratio and the incident stroke in different sensitivity models. [file 13098_2024_1339_MOESM1_ESM.docx]

**Table S1** The characteristics of participants in smoking status.

| Smoking status | Never smokers | Ever smokers or current smokers | P-value |
| --- | --- | --- | --- |
| Participants | 6250 | 3914 |  |
| Gender |  |  | <0.001 |
| Male | 1253 (20.048%) | 3493 (89.244%) |  |
| Female | 4997 (79.952%) | 421 (10.756%) |  |
| Age (years) | 58.68 ± 9.44 | 59.97 ± 9.14 | <0.001 |
| Drinking status |  |  | <0.001 |
| Never drinkers | 587 (9.39%) | 797 (20.36%) |  |
| Ever drinkers | 4906 (78.50%) | 1314 (33.57%) |  |
| Current drinkers | 757 (12.11%) | 1803 (46.07%) |  |
| Physical activity |  |  | 0.003 |
| No | 3972 (63.55%) | 2599 (66.40%) |  |
| Yes | 2278 (36.45%) | 1315 (33.60%) |  |
| SBP (mmHg) | 130.57 ± 21.64 | 130.62 ± 20.85 | 0.919 |
| DBP (mmHg) | 75.58 ± 11.87 | 76.18 ± 12.39 | 0.014 |
| BMI (kg/m^2^) | 23.98 ± 4.03 | 22.80 ± 3.67 | <0.001 |
| LDL-C (mg/dL) | 119.22 ± 34.44 | 113.11 ± 33.45 | <0.001 |
| TC (mg/dL) | 195.36 ± 38.15 | 188.93 ± 37.65 | <0.001 |
| CRP (mg/L) | 0.98 (0.53-2.00) | 1.09 (0.58-2.31) | <0.001 |
| Scr (mg/dL) | 0.73 ± 0.21 | 0.86 ± 0.26 | <0.001 |
| FPG (mg/dL) | 109.51 ± 35.17 | 109.26 ± 34.35 | 0.732 |
| HbA1c (%) | 5.25 ± 0.82 | 5.22 ± 0.76 | 0.023 |
| Cystatin C (mg/L) | 0.97 ± 0.27 | 1.05 ± 0.29 | <0.001 |
| UA(mg/dL) | 4.21 ± 1.17 | 4.82 ± 1.28 | <0.001 |

Values are n (%) or mean ± SD or median (quartile)

SBP: systolic blood pressure; DBP: diastolic blood pressure; BMI: body mass index; HDL-C: high-density lipoprotein cholesterol; LDL-C: low-density lipoprotein cholesterol; TC: total cholesterol; TG: triglycerides; Scr: serum creatinine; CRP: C-reactive protein; FPG: fasting plasma glucose; HbA1c: glycosylated hemoglobin; UA: uric acid

**Table S2** Relationship between TG/HDL-C ratio and the incident stroke in different sensitivity models.

| Exposure | Model 1 (HR.,95% CI, P) | Model 2 (HR,95% CI, P) | Model 3 (HR,95% CI, P) |
| --- | --- | --- | --- |
| TG/HDL-C ratio | 1.06 (1.04, 1.08) <0.0001 | 1.06 (1.04, 1.08) <0.0001 | 1.04 (1.01, 1.06) 0.0037 |
| TG/HDL-C ratio (quartile) |  |  |  |
| Q1 | ref | ref | ref |
| Q2 | 1.27 (1.06, 1.51) 0.0089 | 1.27 (1.06, 1.52) 0.0085 | 1.19 (1.00, 1.43) 0.0547 |
| Q3 | 1.48 (1.25, 1.76) <0.0001 | 1.49 (1.25, 1.77) <0.0001 | 1.30 (1.09, 1.55) 0.0041 |
| Q4 | 1.77 (1.49, 2.09) <0.0001 | 1.81 (1.53, 2.14) <0.0001 | 1.41 (1.18, 1.68) 0.0001 |
| P for trend | <0.0001 | <0.0001 | <0.0001 |

Note: we did not adjust for BMI, physical activity, and Cystatin C in Model 1, Model 2, and Model 3.

Model 1: we did not adjust for other covariants..

Model 2: we adjusted for gender, age, drinking status, and smoking status.

age, gender, educational attainment, annual income, smoking status, and drinking status.

Model 3: we adjusted for gender, age, drinking status, smoking status, drugs for cardiovascular prevention, hypertension, FPG, TC, CRP, LDL-C, Scr, HbA1c, and UA.

HR: hazard ratios; CI: confidence interval; Ref: reference; TG/HDL-C ratio: ratio of triglyceride to high-density lipoprotein cholesterol

**Table S3** Relationship between TG/HDL-C ratio and the incident stroke in different sensitivity models.

| Exposure | Model 1 (HR.,95% CI, P) | Model 2 (HR,95% CI, P) | Model 3 (HR,95% CI, P) |
| --- | --- | --- | --- |
| TC/HDL-C ratio (per 1 increase) | 1.06 (1.04, 1.08) <0.0001 | 1.03 (1.01, 1.05) 0.0030 | 1.03 (1.00, 1.06) 0.0367 |
| TC/HDL-C ratio (quartile) |  |  |  |
| Q1 | ref | ref | ref |
| Q2 | 1.37 (1.12, 1.67) 0.0018 | 1.30 (1.07, 1.59) 0.0095 | 1.29 (1.06, 1.58) 0.0120 |
| Q3 | 1.58 (1.31, 1.92) <0.0001 | 1.37 (1.13, 1.66) 0.0016 | 1.36 (1.12, 1.66) 0.0023 |
| Q4 | 1.96 (1.63, 2.36) <0.0001 | 1.53 (1.26, 1.85) <0.0001 | 1.46 (1.19, 1.78) 0.0002 |
| P for trend | <0.001 | <0.001 | 0.0003 |

Model 1: we did not adjust for other covariants.

Model 2: we adjusted for BMI, gender, age, drinking status, physical activity, and smoking status.

Model 3: we adjusted for BMI, gender, age, drinking status, physical activity, smoking status, drugs for cardiovascular prevention, hypertension, FPG, TC, CRP, LDL-C, Scr, HbA1c, Cystatin C, and UA.

HR: hazard ratios; CI: confidence interval; Ref: reference; TG/HDL-C ratio: ratio of triglyceride to high-density lipoprotein cholesterol
